# General Randomized Response Techniques Using Polya's Urn Process as a Randomization Device

**DOI:** 10.1371/journal.pone.0115612

**Published:** 2014-12-26

**Authors:** Mashail M. Al-Sobhi, Zawar Hussain, Bander Al-Zahrani

**Affiliations:** 1 Department of Mathematics, Umm Alqura University 21421, Makkah, Saudi Arabia; 2 Department of Statistics, Quaid-i-Azam University 45320, Islamabad, Pakistan; 3 Department of Statistics, King Abdulaziz University 80203, Jeddah, Saudi Arabia; National Institute of Genomic Medicine, Mexico

## Abstract

In this paper, interesting improvements in [1] and [2] randomized response techniques have been proposed. The proposed randomized response technique applies Polya's urn process (see [3]) to obtain data from respondents. One of the suggested technique requires reporting the number of draws to observe a fixed number of cards of certain type. On the contrary, the number of cards of a certain type is to be reported in case of second proposed randomized response model. Based on the information collected through the suggested techniques, two different unbiased estimators of proportion of a sensitive attribute have been suggested. A detailed comparative simulation study has also been done. The results are also supported by means of a small scale survey.

## Introduction

Surveys and questionnaires are usual statistical tools for obtaining data about attitudes, behaviors, emotions, and so forth. The important assumption of any survey technique is that the respondents are completely truthful in their reporting. However, the legitimacy of this assumption is dubious when investigators ask questions that most would be hesitant to respond publicly. Examples of such questions are those that disclose whether the respondent possesses an illicit behavior, or a trait that is socially undesirable, or the question may concern with the trait of which respondent is embarrassed or which the respondent feels extremely personal to reveal openly. Faced with such questions, some respondents in a sample will decline to respond or will misreport. Either type of avoidance introduces a bias into collected information. Hence, there are serious procedural hurdles to conduct surveys in studies in which a stigmatizing characteristic is associated with the phenomenon of concern.

To overcome these hurdles [4], developed the randomized response technique (RRT). [4] method is meant for estimating the proportion of a sensitive attribute prevailing in population of interest. It is based on the hypothesis that cooperation by the individuals should recover if their replies would not expose their true status. A number of variations of [4] RRT and new RRTs have been proposed by many researchers like [1], [2], [5], [6], [7], [8], [9], [10], [11], [12], [13], [14], [15], [16], [17], and many others. In most of the RRTs, yielding either qualitative or quantitative response, reported response, commonly, follows either Bernoulli ([4], [18], [1], [9], [10], etc.), Poisson ([16]), multinomial ([8]), geometric ([2]) or negative hypergeometric distribution ([17]).

[1] introduced an ingenious RRT which produces the response following a Bernoulli distribution. A number of RRTs such as the [4], [10], [9] are special cases of [1] technique. [19] have reported that the [1]′s family of RRTs performs better than the Simmon's family in terms of efficiency and privacy protection. Recently [2], improved the [1]′s RRT by introducing geometric distribution as a randomization device.

There is an extensive amount of literature on the applications of urn models in different fields like genetics, capture-recapture models, computer theory, biology, learning processes, etc. Some of applications of urn models in epidemiology are noted by [20], [21] and [22], amongst many others. Applications to botany, lexicology and numismatics are mentioned by [23]. Interested readers may refer to [24] and [25] for a detailed account on the applications of urn models in randomization structures.

As far as RRT is concerned, urn scheme may be applied to determine the questions asked in a survey enquiry. The pioneer RRT proposed by [4], and further extended by [18] and [26] is a striking example. Utilization of urn model as a randomization device for [4] RRT may be briefly explained as follows. In the [4] RRT, the respondent randomly draws a card from an urn containing *g* green and *r* red cards. If a green card is drawn, the respondent will report (*yes* or *no*) to the question, “I am a member of sensitive group”, and replace the card drawn. If a red card is drawn, the question is, “I am not a member of sensitive group”. The interviewer is unaware of the colors of the cards drawn by the respondents, but the probability of drawing a green card is known. Under the assumptions of randomness of the drawing of balls and truthful reporting of answers, obviously, the total number of *yes* responses in a sample of *n* respondents follows a binomial distribution with parameters 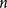 and 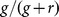.

Of the many urn models, the Polya's urn model is very popular within Statistics because it generalizes the binomial, hypergeometric, and beta-Bernoulli (beta-binomial) distributions through a single formula. In the present study, we intend to apply Polya's urn scheme to randomize the responses. It is important to note that different discrete distributions such as binomial, hypergeometric, negative binomial, geometric, negative hypergeometric, beta binomial, uniform, etc. can be generated through Polya's urn scheme. Thus, using Polya's urn schemes may be taken as more flexible and a generalization of the above mentioned distributions. The idea is, actually, taken from the [2] RRT (a special case of [1] RRT) which yields a response following a geometric distribution. The rest of the paper is organized as follows. In the next section, we present the [1] and [2] RRTs. Two new estimators using Polya's urn process have been suggested in Section 3. Section 4 consists of discussion and conclusion of the study. A real life example has been presented in Section 5.

## Some Recent Related RRTs

In this section, we present brief summaries of the [1] and [2] RRTs and introduce the notations. Let 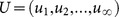 be an infinite dichotomous population and every individual in the population belongs either to a sensitive group (possessing a sensitive attribute) 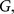 or to its complement 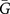. The problem is to estimate 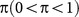, the unknown proportion of population members in group 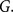 To do so, a sample 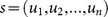 of size 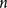 is drawn from the population 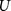 using a simple random sampling with replacement sampling scheme. Because of the sensitive nature of the attribute under study, a direct question regarding membership in 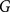 or otherwise is not expected to be helpful in terms of cooperation from the respondents. Thus, an alternative procedure such as RRT is needed if we are to procure reliable data on the sensitive attribute.

Two of the background RRTs are discussed in the following subsections.

### 1.1. Kuk [1] RRT

In this RRT, if a respondent belongs to a sensitive group 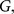 then he/she is instructed to use a deck of cards having 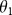 proportion of cards with the statement, “ I 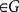
” and if he/she belongs to non-sensitive group 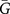, then he/she is requested to use a different deck of cards having 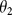 proportion of cards with the statement, “
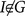
”. The probability of a *yes* response in the [1] model is given by
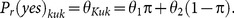
(2.1)


An unbiased estimator of 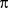 is given by
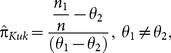
(2.2)where 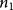 is the observed number of *yes* responses in the sample 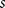 and follows a binomial distribution with parameters 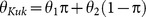 and 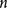. Thus the variance of 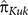 is given by
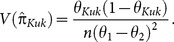
(2.3)


### 1.2. Singh and Grewal [2] RRT

In this RRT, each respondent is provided with two decks of cards in the same way as in the [1] RRT. In the first deck of cards 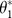 is the proportion of cards with the statement, “I 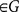
” and 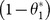 be the proportion of cards with the statement, “
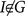
”. In the second deck of cards 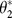 is the proportion of cards with the statement, “I 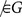
” and 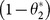 be the proportion of cards with the statement, “
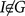
”. Up till here, it is same as that of the [1]. If a respondent belongs to sensitive group 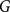, he/she is instructed to draw cards, one by one using with replacement, from the first deck of cards until he/she gets the first card bearing the statement of his/her own status, and requested to report the number of cards, say 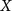, drawn by him/her to obtain the first card of his/her own status. If a respondent belongs to non-sensitive group 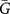, he/she is instructed to draw cards, one by one using with replacement drawing, from the second deck of cards until he/she gets the first card bearing the statement of his/her own status, and requested to report the number of cards, say 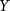, drawn by him/her to obtain the first card of his/her own status. Since cards are drawn using with replacement sampling, it is clear that 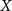 and 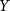 follow geometric distributions with parameters 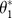 and 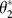, respectively. If 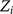 denotes the number of cards reported by the 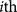 respondent, then it can be written as 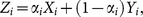
where 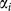 is a Bernoulli random variable with 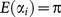. The expectation of reported number of cards is given by
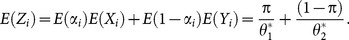
(2.4)


An unbiased estimator of 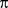 proposed by [2] is given by
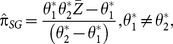
(2.5)with variance given by
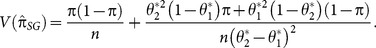
(2.6)


## Proposed RRTs

In this section, we present two new RRTs using Polya's urn scheme.

### 3.1. Proposed RRT I

A more general RRT is explained below. Consider two decks having two types of cards, red and green. The deck 1 contains 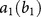 red (green) cards. The deck 2 contains 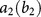 red (green) cards. Each respondent belonging to the sensitive (non-sensitive) group is requested to use deck 1 (deck 2) and randomly draw 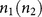 cards one by one. On each draw he/she is requested to replace the card drawn and add 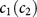 cards of the same color. If a respondent belongs to sensitive (non-sensitive) group he is required to report the number of red cards drawn, say 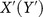, in 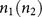 draws. Obviously, here 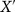 and 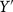 have the distributions 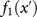 and 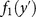, respectively. The functional forms of 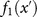 and 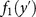 are given by
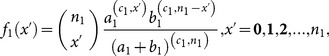
(3.1)

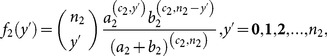
(3.2)where 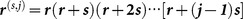 for 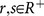 and 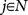.

The response 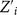 from the 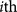 respondent may be written as
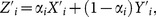
(3.3)where 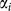 is a random variable defined as above and 
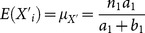
 and 
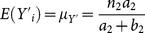
. Thus, expected response may be written as
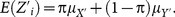
(3.4)


Now an unbiased estimator of population proportion 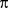 may be defined and its variance can easily be worked out. By solving (3.4) for 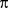 and estimating 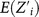 by 
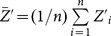
, an unbiased estimator of 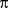 is suggested as follows:
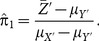
(3.5)


Its variance is given by
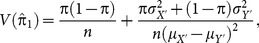
(3.6)where


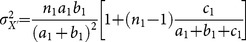
 and 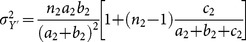.

Following remarks are in order.

**Remark 1:** It is interesting to see that the reported response 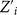 follows a two component mixture distribution with 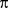 and 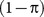 as the mixing probabilities. For 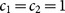, the distribution of response 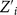 is a mixture of two beta-binomial distributions with parameters 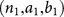 and 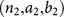.

**Remark 2:** For 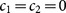, the distribution of 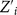 is a mixture of two binomial distributions with parameters 
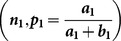
 and 
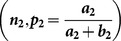
.

**Remark 3:** For 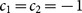, the distribution of 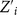 is a mixture of two hypergeometric distributions with parameters 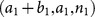 and 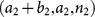. In this case we must have 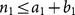 and 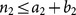.

**Remark 4:** If 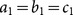 and 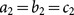, the distribution of 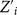 is a mixture of two uniform distributions with parameters 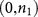 and 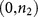.

### 3.2. Proposed RRT II

The proposed RRT II works in a fashion similar to that of Proposed RRT I. Here, we assume that 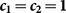, and respondents are requested to report the number of draws to observe a fixed number, say 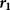 and 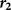, of red cards. Let 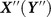 denotes the number of draws from urn 1 (urn 2) required to observe 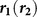 red cards. Obviously, now, 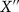 and 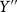 have the distributions given by
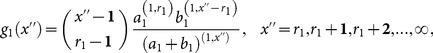
(3.7)

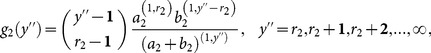
(3.8)


The response 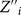 from the 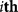 respondent may be written as 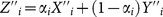, where 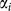 is a random variable defined as above and 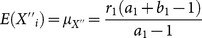 and 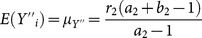. Thus, expected response may be written as
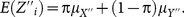
(3.9)


Now, following the steps as in subsection 3.1, an unbiased estimator of population proportion 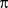 may be defined and its variance can be derived. By solving (3.9) for 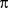 and estimating 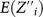 by 
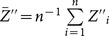
, an unbiased estimator of 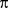 is suggested as
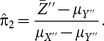
(3.10)


Its variance is given by
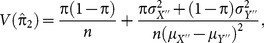
(3.11)where
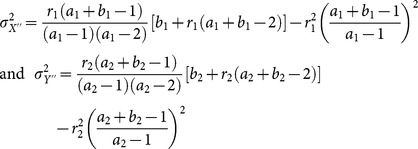



**Remark 5:** For 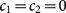, the distribution of 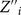 is a mixture of two negative binomial distributions with parameters 
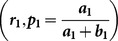
 and 
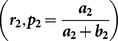
.

**Remark 6:** For 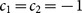, the distribution of 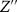 is a mixture of two negative hypergeometric distributions with parameters 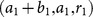 and 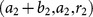.

**Remark 7:** For 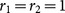, the distribution of 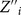 is a mixture of two geometric distributions.

**Remark 8:** If 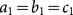 and 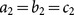, the distribution of 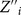 is a mixture of two uniform distributions.

## Discussion and Conclusion

Since our objective in this study was to introduce an application of Polya's urn process to obtain data on sensitive variables, we did not intend to have a full-fledged comparative study of proposed estimators with any other estimators. However, to have an idea, we just considered estimator 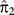 and compared it with [1] and [2] estimators assuming 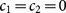, 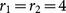, 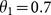 and 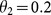. The reason of setting 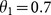 and 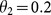 is that the [1] model is at its best when 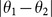 is maximum. As mentioned in Remark 5 above, for 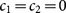, we have 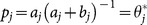 for 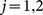. The relative efficiency (RE) of the estimator 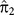 relative to 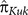 and 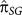 is defined as 
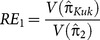
 and 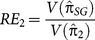, respectively. The RE results are displayed in S1 Table available in the supporting information files. From S1 Table (see S1 Table), it observed that proposed estimator is relatively more efficient than that of [1] and [2]. For the situations, where 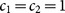 and 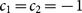, we have done a bit detailed comparison study. As, 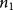 (or 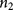) is the fixed number of cards to be drawn in the proposed RRT I and  (or 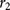) is pre decided number of cards of certain type in proposed RRT II, we fixed 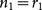 and 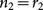 so that proposed estimators could be compared with each other on equal footings. The RE of the proposed estimators 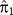 and 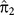 relative to 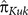 and 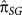 is arranged in the S2–S4 Tables and S5–S10 Tables, respectively. It was observed that RE of proposed estimators relative to 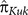 increases with the increase in 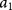 when 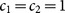 (see S2 and S3 Tables). The proposed estimators are also more efficient when we take 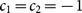 (see S4 Table). Same is the behavior of RE of the proposed estimators when we compare them with 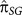 (see S5–S10 Tables). From the S2–S10 Tables (see S2–S10 Tables), it is evident that the proposed estimators outshine the two competing estimators 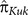 and 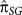. Also, it can be observed that RE of both the estimators is higher (lower) for larger (smaller) 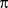 when either 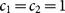 or 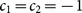, whereas, for 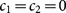, the situation is reversed. The RE of proposed estimators is directly proportional to the difference between 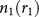 and 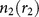. The overall finding is that the proposed estimator 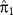 is comparatively more efficient than 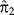. That is, using number of cards of certain type in fixed drawings is more useful than forcing the respondent to keep drawing the cards until he/she observes a pre-decided number of cards of one kind.

It is to be noted that variances 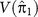 and 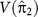 are decreasing functions of 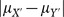 and 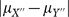, respectively. Thus, variances of the proposed estimators may be cut down to a desired level by suitably choosing the values of 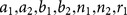 and 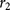 so that 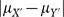 and 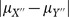 is a maximum.

Moreover, it is seen that the Polya's urn process generates different distributions. Thus, using Polya's urn process is more general and more flexible scheme to generate a randomized response following a desired distribution. Additionally, in the proposed RRTs, no additional sampling cost is needed and every respondent uses the same randomization device. These two features of the proposal may be considered as extra advantages associated with it.

### A practical example

As a practical example, we conducted a small scale survey by drawing a sample of size 100. Consider the population of students currently enrolled in different programs at Quaid-i-Azam University, Islamabad. The students were requested verbally to volunteer themselves for this survey study and were assured that their identity will not be disclosed in anyway. From this population, we took 1000 students including 200 those students who had been using marijuana for the last six months. The purpose of this was to take a population with known population proportion of marijuana users, that is, we took 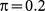. As from the simulation results, it is evident that the proposed RRT 1 is relatively better than the others, therefore, we decided to apply the proposed RRT 1 in actual application. A simple random sample of 100 students (out of 1000 selected students) was drawn using with replacement sampling and every selected student was given two urns each containing red and green cards. The urn 1 (urn 2) contains 1000 (100) red and 300 (40) green cards. For generating data through proposed RRT 1, he/she, then, was asked to draw 3 (3) cards at random from urn 1 (urn 2) if he/she had used (not used) marijuana, at least once, in the last six months. At each draw, he/she was directed to replace two cards (i.e. 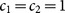) of the color of the card drawn. After drawing the cards, he/she was requested to report the number of red cards. For generating responses through [1] and [2] RRTs, we fixed 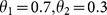 and 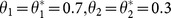. It is to be noted that the same respondents were taken to generate the responses through three different randomization devices considered in this study. The data obtained through these randomization devices are presented in S11–S13 Tables (see S11–S13 Tables). The estimates of the proportion of students who had used marijuana at least once, during the last six months, are obtained as 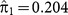, 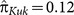 and 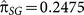. From these estimates, it is clear that the proposed RRT 1 provided the closest estimate of the population proportion, i.e. 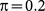. Hence, the proposed RRT 1 is more accurate than the other RRTs considered in this small scale survey.

## Supporting Information

S1 Table

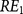
 and 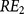 values for 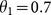, 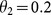 and 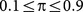.(DOCX)

S2 TableRelative efficiency of 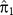 (**in bold**) 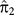 with respect to 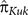 for 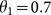, 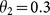, 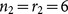, 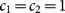, 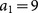, 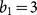, 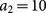, 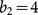.(DOCX)

S3 TableRelative efficiency of 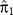 (**in bold**) 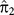 with respect to 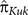 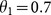, 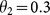, 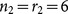, 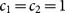, 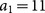, 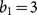, 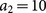, 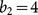,(DOCX)

S4 TableRelative efficiency of 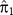 (**in bold**) 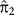 with respect to 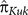 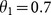, 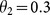, 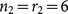, 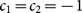, 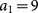, 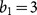, 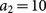, 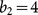,(DOC)

S5 TableRelative efficiency of 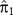
(**in bold**) 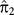 with respect to 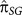 for 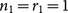, 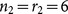, 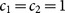, 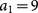, 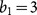, 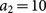, 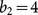, 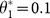.(DOCX)

S6 TableRelative efficiency of 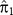 (**in bold**) 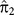 with respect to 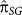 for 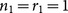, 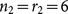, 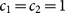, 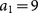, 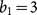, 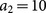, 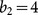, 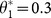.(DOCX)

S7 TableRelative efficiency of 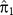 (**in bold**) 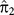 with respect to 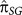 for 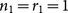, 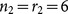, 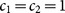, 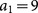, 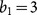, 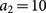, 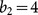, 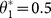.(DOCX)

S8 TableRelative efficiency of 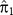 (**in bold**) 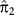 with respect to 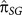 for 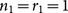, 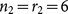, 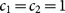, 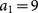, 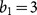, 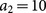, 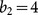, 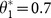.(DOCX)

S9 TableRelative efficiency of 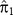 (**in bold**) 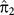 with respect to 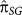 for 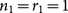, 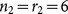, 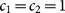, 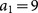, 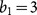, 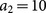, 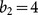, 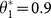.(DOCX)

S10 TableRelative efficiency of 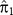 (**in bold**) 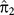 with respect to 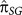 for 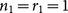, 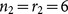, 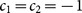, 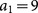, 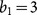, 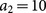, 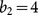, 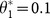.(DOCX)

S11 TableData obtained through proposed RRT 1 using 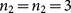, 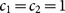, 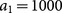, 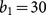, 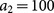, 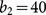.(DOC)

S12 TableData obtained through [1] RRT using 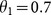, 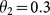.(DOCX)

S13 TableData obtained through [2] RRT using 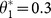, 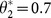.(DOCX)
