# Supplementary material for: General Randomized Response Techniques Using Polya's Urn Process as a Randomization Device
Source: PLoS One. 2014 Dec 26;9(12):e115612. doi: 10.1371/journal.pone.0115612 (PMC4277314; doi:10.1371/journal.pone.0115612)
Supplement: S1 Table — and values for , and . (DOCX) [file pone.0115612.s001.docx]

**Table S1:** $RE_{1}$and $RE_{2}$values for $\theta_{1}=0.7$, $\theta_{2}=0.2$and $0.1\leq\pi\leq0.9$.

| $\pi$ | $\theta_{1}^{*}$ | $\theta_{2}^{*}$ | $RE_{1}$ | $RE_{2}$ | $\pi$ | $\theta_{1}^{*}$ | $\theta_{2}^{*}$ | $RE_{1}$ | $RE_{2}$ |
| --- | --- | --- | --- | --- | --- | --- | --- | --- | --- |
| 0.1 | 0.1 | 0.2 | 2.083 | 3.250 | 0.2 | 0.1 | 0.2 | 1.680 | 3.040 |
|  |  | 0.3 | 4.167 | 2.500 |  |  | 0.3 | 2.835 | 2.380 |
|  |  | 0.4 | 5.172 | 2.138 |  |  | 0.4 | 3.316 | 2.105 |
|  |  | 0.5 | 5.674 | 1.957 |  |  | 0.5 | 3.551 | 1.971 |
|  |  | 0.6 | 5.952 | 1.857 |  |  | 0.6 | 3.684 | 1.895 |
|  |  | 0.7 | 6.122 | 1.796 |  |  | 0.7 | 3.768 | 1.847 |
|  |  | 0.8 | 6.234 | 1.756 |  |  | 0.8 | 3.825 | 1.814 |
|  |  | 0.9 | 6.312 | 1.728 |  |  | 0.9 | 3.856 | 1.791 |
|  | 0.2 | 0.3 | 0.833 | 3.700 |  | 0.2 | 0.3 | 0.778 | 3.556 |
|  |  | 0.4 | 2.459 | 3.115 |  |  | 0.4 | 1.909 | 2.909 |
|  |  | 0.5 | 3.835 | 2.619 |  |  | 0.5 | 2.662 | 2.479 |
|  |  | 0.6 | 4.762 | 2.286 |  |  | 0.6 | 3.111 | 2.222 |
|  |  | 0.7 | 5.357 | 2.071 |  |  | 0.7 | 3.387 | 2.565 |
|  |  | 0.8 | 5.745 | 1.932 |  |  | 0.8 | 3.566 | 1.962 |
|  |  | 0.9 | 6.005 | 1.838 |  |  | 0.9 | 3.688 | 1.892 |
|  | 0.3 | 0.4 | 0.473 | 3.830 |  | 0.3 | 0.4 | 0.467 | 3.733 |
|  |  | 0.5 | 1.657 | 3.403 |  |  | 0.5 | 1.391 | 3.205 |
|  |  | 0.6 | 3.000 | 2.920 |  |  | 0.6 | 2.211 | 2.737 |
|  |  | 0.7 | 4.131 | 2.513 |  |  | 0.7 | 2.791 | 2.405 |
|  |  | 0.8 | 4.967 | 2.212 |  |  | 0.8 | 3.182 | 2.182 |
|  |  | 0.9 | 5.556 | 2.000 |  |  | 0.9 | 3.446 | 2.031 |
|  | 0.4 | 0.5 | 0.331 | 3.881 |  | 0.4 | 0.5 | 0.335 | 3.809 |
|  |  | 0.6 | 1.282 | 3.538 |  |  | 0.6 | 1.120 | 3.360 |
|  |  | 0.7 | 2.571 | 3.074 |  |  | 0.7 | 1.953 | 2.884 |
|  |  | 0.8 | 3.846 | 2.615 |  |  | 0.8 | 2.625 | 2.500 |
|  |  | 0.9 | 4.902 | 2.235 |  |  | 0.9 | 3.111 | 2.222 |
|  | 0.5 | 0.6 | 0.269 | 3.903 |  | 0.5 | 0.6 | 0.275 | 3.843 |
|  |  | 0.7 | 1.128 | 3.594 |  |  | 0.7 | 0.999 | 3.429 |
|  |  | 0.8 | 2.468 | 3.112 |  |  | 0.8 | 1.871 | 2.931 |
|  |  | 0.9 | 3.980 | 2.507 |  |  | 0.9 | 2.643 | 2.490 |
|  | 0.6 | 0.7 | 0.249 | 3.910 |  | 0.6 | 0.7 | 0.255 | 3.855 |
|  |  | 0.8 | 1.45 | 3.558 |  |  | 0.8 | 1.000 | 3.429 |
|  |  | 0.9 | 2.778 | 3.000 |  |  | 0.9 | 2.000 | 2.857 |
|  | 0.7 | 0.8 | 0.270 | 3.903 |  | 0.7 | 0.8 | 0.273 | 3.844 |
|  |  | 0.9 | 1.449 | 3.478 |  |  | 0.9 | 1.185 | 3.323 |
|  | 0.8 | 0.9 | 0.388 | 3.860 |  | 0.8 | 0.9 | 0.373 | 3.787 |

(Table S1 continued)

| $\pi$ | $\theta_{1}^{*}$ | $\theta_{2}^{*}$ | $RE_{1}$ | $RE_{2}$ | $\pi$ | $\theta_{1}^{*}$ | $\theta_{2}^{*}$ | $RE_{1}$ | $RE_{2}$ |
| --- | --- | --- | --- | --- | --- | --- | --- | --- | --- |
| 0.3 | 0.1 | 0.2 | 1.468 | 2.984 | 0.4 | 0.1 | 0.2 | 1.333 | 3.000 |
|  |  | 0.3 | 2.318 | 2.395 |  |  | 0.3 | 2.018 | 2.464 |
|  |  | 0.4 | 2.663 | 2.156 |  |  | 0.4 | 2.341 | 2.241 |
|  |  | 0.5 | 2.875 | 2.037 |  |  | 0.5 | 2.491 | 2.131 |
|  |  | 0.6 | 2.935 | 1.968 |  |  | 0.6 | 2.581 | 2.065 |
|  |  | 0.7 | 3.000 | 1.923 |  |  | 0.7 | 2.639 | 2.021 |
|  |  | 0.8 | 3.045 | 1.892 |  |  | 0.8 | 2.680 | 1.990 |
|  |  | 0.9 | 3.077 | 1.869 |  |  | 0.9 | 2.711 | 1.967 |
|  | 0.2 | 0.3 | 0.734 | 3.492 |  | 0.2 | 0.3 | 0.696 | 3.478 |
|  |  | 0.4 | 1.640 | 2.865 |  |  | 0.4 | 1.477 | 2.892 |
|  |  | 0.5 | 2.190 | 2.484 |  |  | 0.5 | 1.937 | 2.547 |
|  |  | 0.6 | 2.510 | 2.262 |  |  | 0.6 | 2.207 | 2.345 |
|  |  | 0.7 | 2.708 | 2.125 |  |  | 0.7 | 2.376 | 2.218 |
|  |  | 0.8 | 2.839 | 2.035 |  |  | 0.8 | 2.490 | 2.133 |
|  |  | 0.9 | 2.930 | 1.972 |  |  | 0.9 | 2.570 | 2.072 |
|  | 0.3 | 0.4 | 0.456 | 3.684 |  | 0.3 | 0.4 | 0.442 | 3.668 |
|  |  | 0.5 | 1.238 | 3.143 |  |  | 0.5 | 1.134 | 3.149 |
|  |  | 0.6 | 1.857 | 2.714 |  |  | 0.6 | 1.655 | 2.759 |
|  |  | 0.7 | 2.273 | 2.426 |  |  | 0.7 | 2.001 | 2.499 |
|  |  | 0.8 | 2.549 | 2.235 |  |  | 0.8 | 2.233 | 2.326 |
|  |  | 0.9 | 2.737 | 2.105 |  |  | 0.9 | 2.393 | 2.206 |
|  | 0.4 | 0.5 | 0.333 | 3.770 |  | 0.4 | 0.5 | 0.327 | 3.755 |
|  |  | 0.6 | 1.017 | 3.296 |  |  | 0.6 | 0.941 | 3.294 |
|  |  | 0.7 | 1.660 | 2.851 |  |  | 0.7 | 1.485 | 3.887 |
|  |  | 0.8 | 2.141 | 2.518 |  |  | 0.8 | 1.882 | 2.588 |
|  |  | 0.9 | 2.480 | 2.283 |  |  | 0.9 | 2.162 | 2.378 |
|  | 0.5 | 0.6 | 0.275 | 3.810 |  | 0.5 | 0.6 | 0.271 | 3.797 |
|  |  | 0.7 | 0.912 | 3.368 |  |  | 0.7 | 0.847 | 3.365 |
|  |  | 0.8 | 1.586 | 2.902 |  |  | 0.8 | 1.414 | 2.939 |
|  |  | 0.9 | 2.525 | 2.525 |  |  | 0.9 | 1.858 | 2.606 |
|  | 0.6 | 0.7 | 0.255 | 3.824 |  | 0.6 | 0.7 | 0.257 | 3.812 |
|  |  | 0.8 | 0.905 | 3.373 |  |  | 0.8 | 0.835 | 3.374 |
|  |  | 0.9 | 1.655 | 2.855 |  |  | 0.9 | 1.455 | 2.909 |
|  | 0.7 | 0.8 | 0.270 | 3.813 |  | 0.7 | 0.8 | 0.264 | 3.802 |
|  |  | 0.9 | 1.034 | 3.284 |  |  | 0.9 | 0.931 | 3.302 |
|  | 0.8 | 0.9 | 0.358 | 3.752 |  | 0.8 | 0.9 | 0.340 | 3.745 |

(Table S1 continued)

| $\pi$ | $\theta_{1}^{*}$ | $\theta_{2}^{*}$ | $RE_{1}$ | $RE_{2}$ | $\pi$ | $\theta_{1}^{*}$ | $\theta_{2}^{*}$ | $RE_{1}$ | $RE_{2}$ |
| --- | --- | --- | --- | --- | --- | --- | --- | --- | --- |
| 0.5 | 0.1 | 0.2 | 1.238 | 3.063 | 0.6 | 0.1 | 0.2 | 1.163 | 3.163 |
|  |  | 0.3 | 1.886 | 3.571 |  |  | 0.3 | 1.782 | 2.717 |
|  |  | 0.4 | 2.160 | 2.364 |  |  | 0.4 | 2.035 | 2.521 |
|  |  | 0.5 | 2.304 | 2.255 |  |  | 0.5 | 2.202 | 2.414 |
|  |  | 0.6 | 2.391 | 2.188 |  |  | 0.6 | 2.294 | 2.349 |
|  |  | 0.7 | 2.449 | 2.144 |  |  | 0.7 | 2.355 | 2.304 |
|  |  | 0.8 | 2.491 | 2.113 |  |  | 0.8 | 2.400 | 2.272 |
|  |  | 0.9 | 2.522 | 2.090 |  |  | 0.9 | 2.433 | 2.248 |
|  | 0.2 | 0.3 | 0.660 | 3.500 |  | 0.2 | 0.3 | 0.625 | 3.500 |
|  |  | 0.4 | 1.366 | 2.966 |  |  | 0.4 | 1.282 | 3.033 |
|  |  | 0.5 | 1.782 | 2.650 |  |  | 0.5 | 1.679 | 2.791 |
|  |  | 0.6 | 2.031 | 2.462 |  |  | 0.6 | 1.923 | 2.615 |
|  |  | 0.7 | 2.190 | 2.391 |  |  | 0.7 | 2.083 | 2.500 |
|  |  | 0.8 | 2.299 | 2.258 |  |  | 0.8 | 2.195 | 2.420 |
|  |  | 0.9 | 2.378 | 2.199 |  |  | 0.9 | 2.277 | 2.361 |
|  | 0.3 | 0.4 | 0.426 | 3.677 |  | 0.3 | 0.4 | 0.407 | 3.707 |
|  |  | 0.5 | 1.056 | 3.200 |  |  | 0.5 | 0.991 | 3.286 |
|  |  | 0.6 | 1.523 | 2.846 |  |  | 0.6 | 1.429 | 2.971 |
|  |  | 0.7 | 1.837 | 2.609 |  |  | 0.7 | 1.729 | 2.755 |
|  |  | 0.8 | 2.050 | 2.447 |  |  | 0.8 | 1.938 | 2.609 |
|  |  | 0.9 | 2.200 | 2.737 |  |  | 0.9 | 2.089 | 2.496 |
|  | 0.4 | 0.5 | 0.317 | 3.760 |  | 0.4 | 0.5 | 0.304 | 3.781 |
|  |  | 0.6 | 0.880 | 3.333 |  |  | 0.6 | 0.826 | 3.405 |
|  |  | 0.7 | 1.366 | 2.966 |  |  | 0.7 | 1.277 | 3.081 |
|  |  | 0.8 | 1.722 | 2.696 |  |  | 0.8 | 1.613 | 2.839 |
|  |  | 0.9 | 1.976 | 2.503 |  |  | 0.9 | 1.859 | 2.062 |
|  | 0.5 | 0.6 | 0.264 | 3.800 |  | 0.5 | 0.6 | 0.254 | 3.817 |
|  |  | 0.7 | 0.792 | 3.400 |  |  | 0.7 | 0.743 | 3.465 |
|  |  | 0.8 | 1.296 | 3.018 |  |  | 0.8 | 1.206 | 3.131 |
|  |  | 0.9 | 1.690 | 2.720 |  |  | 0.9 | 1.574 | 2.867 |
|  | 0.6 | 0.7 | 0.244 | 3.815 |  | 0.6 | 0.7 | 0.235 | 3.831 |
|  |  | 0.8 | 0.766 | 3.412 |  |  | 0.8 | 0.725 | 3.478 |
|  |  | 0.9 | 1.320 | 3.000 |  |  | 0.9 | 1.220 | 3.122 |
|  | 0.7 | 0.8 | 0.255 | 3.806 |  | 0.7 | 0.8 | 0.244 | 3.824 |
|  |  | 0.9 | 0.852 | 3.355 |  |  | 0.9 | 0.785 | 3.435 |
|  | 0.8 | 0.9 | 0.322 | 3.756 |  | 0.8 | 0.9 | 0.302 | 3.782 |

(Table S1 continued)

| $\pi$ | $\theta_{1}^{*}$ | $\theta_{2}^{*}$ | $RE_{1}$ | $RE_{2}$ | $\pi$ | $\theta_{1}^{*}$ | $\theta_{2}^{*}$ | $RE_{1}$ | $RE_{2}$ |
| --- | --- | --- | --- | --- | --- | --- | --- | --- | --- |
| 0.7 | 0.1 | 0.2 | 1.100 | 3.300 | 0.8 | 0.1 | 0.2 | 1.043 | 3.478 |
|  |  | 0.3 | 1.714 | 2.909 |  |  | 0.3 | 1.673 | 3.163 |
|  |  | 0.4 | 2.000 | 2.727 |  |  | 0.4 | 1.986 | 3.007 |
|  |  | 0.5 | 2.160 | 2.626 |  |  | 0.5 | 2.168 | 2.916 |
|  |  | 0.6 | 2.260 | 2.562 |  |  | 0.6 | 2.286 | 2.857 |
|  |  | 0.7 | 2.329 | 2.518 |  |  | 0.7 | 2.368 | 2.816 |
|  |  | 0.8 | 2.380 | 2.486 |  |  | 0.8 | 2.428 | 2.786 |
|  |  | 0.9 | 2.418 | 2.461 |  |  | 0.9 | 2.475 | 2.763 |
|  | 0.2 | 0.3 | 0.289 | 3.625 |  | 0.2 | 0.3 | 0.552 | 3.724 |
|  |  | 0.4 | 0.215 | 3.227 |  |  | 0.4 | 1.157 | 3.422 |
|  |  | 0.5 | 1.608 | 2.977 |  |  | 0.5 | 1.560 | 3.220 |
|  |  | 0.6 | 1.859 | 2.817 |  |  | 0.6 | 1.829 | 3.086 |
|  |  | 0.7 | 2.029 | 2.709 |  |  | 0.7 | 2.017 | 2.992 |
|  |  | 0.8 | 2.150 | 2.632 |  |  | 0.8 | 2.155 | 2.923 |
|  |  | 0.9 | 2.240 | 2.575 |  |  | 0.9 | 2.259 | 2.870 |
|  | 0.3 | 0.4 | 0.384 | 3.755 |  | 0.3 | 0.4 | 0.360 | 3.820 |
|  |  | 0.5 | 0.934 | 3.406 |  |  | 0.5 | 0.886 | 3.560 |
|  |  | 0.6 | 1.356 | 3.137 |  |  | 0.6 | 1.297 | 3.351 |
|  |  | 0.7 | 1.656 | 2.946 |  |  | 0.7 | 1.608 | 3.196 |
|  |  | 0.8 | 1.871 | 2.809 |  |  | 0.8 | 1.839 | 3.080 |
|  |  | 0.9 | 2.031 | 2.708 |  |  | 0.9 | 2.016 | 2.992 |
|  | 0.4 | 0.5 | 0.288 | 3.317 |  | 0.4 | 0.5 | 0.270 | 3.865 |
|  |  | 0.6 | 0.776 | 3.506 |  |  | 0.6 | 0.727 | 3.636 |
|  |  | 0.7 | 1.205 | 3.233 |  |  | 0.7 | 1.143 | 3.429 |
|  |  | 0.8 | 1.535 | 3.023 |  |  | 0.8 | 1.477 | 3.262 |
|  |  | 0.9 | 1.784 | 2.865 |  |  | 0.9 | 1.739 | 3.130 |
|  | 0.5 | 0.6 | 0.241 | 3.847 |  | 0.5 | 0.6 | 0.255 | 3.887 |
|  |  | 0.7 | 0.696 | 3.557 |  |  | 0.7 | 0.649 | 3.675 |
|  |  | 0.8 | 1.133 | 3.279 |  |  | 0.8 | 1.068 | 3.466 |
|  |  | 0.9 | 1.489 | 3.052 |  |  | 0.9 | 1.423 | 3.288 |
|  | 0.6 | 0.7 | 0.222 | 3.858 |  | 0.6 | 0.7 | 0.208 | 3.896 |
|  |  | 0.8 | 0.676 | 3.570 |  |  | 0.8 | 0.627 | 3.686 |
|  |  | 0.9 | 1.138 | 3.276 |  |  | 0.9 | 1.067 | 3.467 |
|  | 0.7 | 0.8 | 0.230 | 3.854 |  | 0.7 | 0.8 | 0.219 | 3.893 |
|  |  | 0.9 | 0.725 | 3.538 |  |  | 0.9 | 0.668 | 3.666 |
|  | 0.8 | 0.9 | 0.281 | 3.821 |  | 0.8 | 0.9 | 0.258 | 3.871 |

(Table S1 continued)

| $\pi$ | $\theta_{1}^{*}$ | $\theta_{2}^{*}$ | $RE_{1}$ | $RE_{2}$ | $\pi$ | $\theta_{1}^{*}$ | $\theta_{2}^{*}$ | $RE_{1}$ | $RE_{2}$ |
| --- | --- | --- | --- | --- | --- | --- | --- | --- | --- |
| 0.9 | 0.1 | 0.2 | 0.989 | 3.707 | 0.9 | 0.3 | 0.7 | 1.578 | 3.532 |
|  |  | 0.3 | 1.655 | 3.509 |  |  | 0.8 | 1.838 | 3.455 |
|  |  | 0.4 | 2.015 | 3.402 |  |  | 0.9 | 2.045 | 3.393 |
|  |  | 0.5 | 2.235 | 3.337 |  | 0.4 | 0.5 | 0.248 | 3.926 |
|  |  | 0.6 | 2.382 | 3.293 |  |  | 0.6 | 0.677 | 3.729 |
|  |  | 0.7 | 2.487 | 3.263 |  |  | 0.7 | 1.085 | 3.678 |
|  |  | 0.8 | 2.506 | 3.239 |  |  | 0.8 | 1.433 | 3.575 |
|  |  | 0.9 | 2.628 | 3.220 |  |  | 0.9 | 1.720 | 3.470 |
|  | 0.2 | 0.3 | 0.511 | 3.848 |  | 0.5 | 0.6 | 0.207 | 3.938 |
|  |  | 0.4 | 1.103 | 3.673 |  |  | 0.7 | 0.601 | 3.822 |
|  |  | 0.5 | 1.528 | 3.547 |  |  | 0.8 | 1.007 | 3.701 |
|  |  | 0.6 | 1.829 | 3.457 |  |  | 0.9 | 1.372 | 3.593 |
|  |  | 0.7 | 2.050 | 3.392 |  | 0.6 | 0.7 | 0.191 | 3.943 |
|  |  | 0.8 | 2.217 | 3.342 |  |  | 0.8 | 0.578 | 3.829 |
|  |  | 0.9 | 2.347 | 3.304 |  |  | 0.9 | 1.000 | 3.703 |
|  | 0.3 | 0.4 | 0.332 | 3.907 |  | 0.7 | 0.8 | 0.195 | 3.942 |
|  |  | 0.5 | 0.825 | 3.755 |  |  | 0.9 | 0.612 | 3.818 |
|  |  | 0.6 | 1.247 | 3.630 |  | 0.8 | 0.9 | 0.234 | 3.931 |
