# Supplementary material for: General Randomized Response Techniques Using Polya's Urn Process as a Randomization Device
Source: PLoS One. 2014 Dec 26;9(12):e115612. doi: 10.1371/journal.pone.0115612 (PMC4277314; doi:10.1371/journal.pone.0115612)
Supplement: S3 Table — Relative efficiency of (in bold) with respect to , , , , , , , , (DOCX) [file pone.0115612.s003.docx]

**Table S3:** Relative efficiency of $\hat{\pi}_{1}$(**in bold**) $\hat{\pi}_{2}$ with respect to $\hat{\pi}_{Kuk}$ $\theta_{1}=0.7$, $\theta_{2}=0.3$,$n_{2}=r_{2}=6$, $c_{1}=c_{2}=1$, $a_{1}=11$, $b_{1}=3$,$a_{2}=10$, $b_{2}=4$,

| $\pi$ | | | | | | | | |
| --- | --- | --- | --- | --- | --- | --- | --- | --- |
| 0.1 | 0.2 | 0.3 | 0.4 | 0.5 | 0.6 | 0.7 | 0.8 | 0.9 |
| $n_{1}=r_{1}=1$ | | | | | | | | |
| **6.637** | **5.466** | **4.953** | **4.770** | **4.830** | **5.148** | **5.865** | **7.450** | **12.122** |
| 6.658 | 5.490 | 4.979 | 4.801 | 4.869 | 5.201 | 5.946 | 7.604 | 12.611 |
| $n_{1}=r_{1}=2$ | | | | | | | | |
| **4.765** | **4.243** | **4.009** | **3.956** | **4.057** | **4.338** | **4.902** | **6.052** | **8.988** |
| 5.205 | 4.564 | 4.279 | 4.207 | 4.313 | 4.628 | 5.275 | 6.637 | 10.385 |
| $n_{1}=r_{1}=3$ | | | | | | | | |
| **2.802** | **2.717** | **2.702** | **2.748** | **2.859** | **3.057** | **3.388** | **3.970** | **5.148** |
| 3.657 | 3.431 | 3.347 | 3.372 | 3.502 | 3.770 | 4.260 | 5.202 | 7.431 |
| $n_{1}=r_{1}=4$ | | | | | | | | |
| **1.098** | **1.147** | **1.198** | **1.255** | **1.319** | **1.397** | **1.495** | **1.627** | **1.817** |
| 2.154 | 2.162 | 2.203 | 2.279 | 2.399 | 2.581 | 2.864 | 3.333 | 4.227 |
