# Supplementary material for: General Randomized Response Techniques Using Polya's Urn Process as a Randomization Device
Source: PLoS One. 2014 Dec 26;9(12):e115612. doi: 10.1371/journal.pone.0115612 (PMC4277314; doi:10.1371/journal.pone.0115612)
Supplement: S4 Table — Relative efficiency of (in bold) with respect to , , , , , , , , (DOC) [file pone.0115612.s004.doc]

**Table S4:** Relative efficiency of $\hat{\pi}_{1}$(**in bold**) $\hat{\pi}_{2}$ with respect to $\hat{\pi}_{Kuk}$ $\theta_{1}=0.7$, $\theta_{2}=0.3$,$n_{2}=r_{2}=6$, $c_{1}=c_{2}=-1$, $a_{1}=9$, $b_{1}=3$,$a_{2}=10$, $b_{2}=4$,

| $\pi$ | | | | | | | | |
| --- | --- | --- | --- | --- | --- | --- | --- | --- |
| 0.1 | 0.2 | 0.3 | 0.4 | 0.5 | 0.6 | 0.7 | 0.8 | 0.9 |
| $n_{1}=r_{1}=1$ | | | | | | | | |
| **8.936** | **6.658** | **5.737** | **5.360** | **5.317** | **5.583** | **6.283** | **7.894** | **12.661** |
| 6.569 | 5.430 | 4.930 | 4.755 | 4.820 | 5.144 | 5.868 | 7.469 | 12.216 |
| $n_{1}=r_{1}=2$ | | | | | | | | |
| **7.019** | **5.609** | **4.989** | **4.735** | **4.727** | **4.957** | **5.518** | **6.737** | **9.929** |
| 5.011 | 4.416 | 4.148 | 4.079 | 4.176 | 4.464 | 5.052 | 6.268 | 9.444 |
| $n_{1}=r_{1}=3$ | | | | | | | | |
| **4.698** | **4.117** | **3.836** | **3.732** | **3.763** | **3.937** | **4.306** | **5.030** | **6.619** |
| 3.364 | 3.176 | 3.104 | 3.122 | 3.228 | 3.444 | 3.830 | 4.540 | 6.069 |
| $n_{1}=r_{1}=4$ | | | | | | | | |
| **2.270** | **2.211** | **2.194** | **2.212** | **2.265** | **2.360** | **2.513** | **2.758** | **3.173** |
| 1.813 | 1.825 | 1.857 | 1.909 | 1.987 | 2.099 | 2.263 | 2.512 | 2.926 |
