# Supplementary material for: General Randomized Response Techniques Using Polya's Urn Process as a Randomization Device
Source: PLoS One. 2014 Dec 26;9(12):e115612. doi: 10.1371/journal.pone.0115612 (PMC4277314; doi:10.1371/journal.pone.0115612)
Supplement: S5 Table — Relative efficiency of (in bold) with respect to for , , , , , , , . (DOCX) [file pone.0115612.s005.docx]

**Table S5:** Relative efficiency of $\hat{\pi}_{1}$(**in bold**) $\hat{\pi}_{2}$ with respect to $\hat{\pi}_{SG}$ for $n_{1}=r_{1}=1$,$n_{2}=r_{2}=6$, $c_{1}=c_{2}=1$, $a_{1}=9$, $b_{1}=3$,$a_{2}=10$, $b_{2}=4$, $\theta_{1}^{*}=0.1$.

| $\pi$ | | | | | | | | |
| --- | --- | --- | --- | --- | --- | --- | --- | --- |
| 0.1 | 0.2 | 0.3 | 0.4 | 0.5 | 0.6 | 0.7 | 0.8 | 0.9 |
| $\theta_{2}^{*}=0.2$ | | | | | | | | |
| **1.747** | **2.269** | **2.735** | **3.237** | **3.859** | **4.724** | **6.090** | **8.688** | **15.825** |
| 2.108 | 2.600 | 3.044 | 3.538 | 4.165 | 5.053 | 6.475 | 9.207 | 16.811 |
| $\theta_{2}^{*}=0.4$ | | | | | | | | |
| **1.203** | **1.717** | **2.143** | **2.579** | **3.097** | **3.800** | **4.893** | **6.956** | **12.600** |
| 1.452 | 1.967 | 2.385 | 2.818 | 3.342 | 4.064 | 5.203 | 7.372 | 13.385 |
| $\theta_{2}^{*}=0.6$ | | | | | | | | |
| **0.908** | **1.391** | **1.775** | **2.153** | **2.590** | **3.172** | **4.067** | **5.744** | **10.315** |
| 1.096 | 1.593 | 1.975 | 2.352 | 2.795 | 3.393 | 4.324 | 6.087 | 10.958 |
| $\theta_{2}^{*}=0.8$ | | | | | | | | |
| 0.820 | 1.282 | 1.645 | 1.998 | 2.401 | 2.933 | 3.748 | 5.271 | 9.416 |
| **0.989** | **1.469** | **1.831** | **2.183** | **2.591** | **3.137** | **3.985** | **5.586** | **10.003** |
