# Supplementary material for: General Randomized Response Techniques Using Polya's Urn Process as a Randomization Device
Source: PLoS One. 2014 Dec 26;9(12):e115612. doi: 10.1371/journal.pone.0115612 (PMC4277314; doi:10.1371/journal.pone.0115612)
Supplement: S6 Table — Relative efficiency of (in bold) with respect to for , , , , , , , . (DOCX) [file pone.0115612.s006.docx]

**Table S6:** Relative efficiency of $\hat{\pi}_{1}$(**in bold**) $\hat{\pi}_{2}$ with respect to $\hat{\pi}_{SG}$ $n_{1}=r_{1}=1$,$n_{2}=r_{2}=6$, $c_{1}=c_{2}=1$, $a_{1}=9$, $b_{1}=3$,$a_{2}=10$, $b_{2}=4$, $\theta_{1}^{*}=0.3$.

| $\pi$ | | | | | | | | |
| --- | --- | --- | --- | --- | --- | --- | --- | --- |
| 0.1 | 0.2 | 0.3 | 0.4 | 0.5 | 0.6 | 0.7 | 0.8 | 0.9 |
| $\theta_{2}^{*}=0.2$ | | | | | | | | |
| **26.588** | **20.858** | **17.718** | **15.921** | **15.009** | **14.868** | **15.695** | **18.380** | **27.304** |
| 32.084 | 23.897 | 19.720 | 17.397 | 16.196 | 15.904 | 16.687 | 19.478 | 29.006 |
| $\theta_{2}^{*}=0.4$ | | | | | | | | |
| **23.560** | **21.630** | **21.384** | **22.312** | **24.443** | **28.249** | **35.052** | **48.823** | **87.817** |
| 28.431 | 24.782 | 23.800 | 24.381 | 26.376 | 30.217 | 37.267 | 51.739 | 93.288 |
| $\theta_{2}^{*}=0.6$ | | | | | | | | |
| **2.833** | **3.348** | **3.869** | **4.485** | **5.289** | **6.443** | **8.301** | **11.871** | **21.729** |
| 3.419 | 3.835 | 4.307 | 4.901 | 5.707 | 6.892 | 8.825 | 12.580 | 23.082 |
| $\theta_{2}^{*}=0.8$ | | | | | | | | |
| **1.296** | **1.854** | **2.322** | **2.803** | **3.379** | **4.163** | **5.386** | **7.697** | **14.021** |
| 1.564 | 2.124 | 2.584 | 3.063 | 3.646 | 4.453 | 5.727 | 8.157 | 14.895 |
