# Supplementary material for: General Randomized Response Techniques Using Polya's Urn Process as a Randomization Device
Source: PLoS One. 2014 Dec 26;9(12):e115612. doi: 10.1371/journal.pone.0115612 (PMC4277314; doi:10.1371/journal.pone.0115612)
Supplement: S7 Table — Relative efficiency of (in bold) with respect to for , , , , , , , . (DOCX) [file pone.0115612.s007.docx]

**Table S7:** Relative efficiency of $\hat{\pi}_{1}$(**in bold**) $\hat{\pi}_{2}$ with respect to $\hat{\pi}_{SG}$ $n_{1}=r_{1}=1$,$n_{2}=r_{2}=6$, $c_{1}=c_{2}=1$, $a_{1}=9$, $b_{1}=3$,$a_{2}=10$, $b_{2}=4$, $\theta_{1}^{*}=0.5$.

| $\pi$ | | | | | | | | |
| --- | --- | --- | --- | --- | --- | --- | --- | --- |
| 0.1 | 0.2 | 0.3 | 0.4 | 0.5 | 0.6 | 0.7 | 0.8 | 0.9 |
| $\theta_{2}^{*}=0.2$ | | | | | | | | |
| **8.198** | **6.380** | **5.331** | **4.659** | **4.209** | **3.910** | **3.742** | **3.744** | **4.200** |
| 9.893 | 7.310 | 5.933 | 5.091 | 4.542 | 4.182 | 3.978 | 3.968 | 4.462 |
| $\theta_{2}^{*}=0.4$ | | | | | | | | |
| **55.854** | **44.290** | **38.142** | **34.870** | **33.591** | **34.197** | **37.372** | **45.760** | **72.074** |
| 67.400 | 50.744 | 42.451 | 38.102 | 36.248 | 36.579 | 39.733 | 48.493 | 76.564 |
| $\theta_{2}^{*}=0.6$ | | | | | | | | |
| **42.269** | **37.853** | **36.687** | **37.673** | **40.738** | **46.587** | **57.308** | **79.266** | **141.770** |
| 51.007 | 43.369 | 40.832 | 41.165 | 43.961 | 49.832 | 60.930 | 84.000 | 150.602 |
| $\theta_{2}^{*}=0.8$ | | | | | | | | |
| **3.670** | **4.234** | **4.846** | **5.594** | **6.591** | **8.040** | **10.387** | **14.913** | **27.432** |
| 4.429 | 4.852 | 5.393 | 6.112 | 7.113 | 8.600 | 11.043 | 15.804 | 29.141 |
