# Supplementary material for: General Randomized Response Techniques Using Polya's Urn Process as a Randomization Device
Source: PLoS One. 2014 Dec 26;9(12):e115612. doi: 10.1371/journal.pone.0115612 (PMC4277314; doi:10.1371/journal.pone.0115612)
Supplement: S8 Table — Relative efficiency of (in bold) with respect to for , , , , , , , . (DOCX) [file pone.0115612.s008.docx]

**Table S8:** Relative efficiency of $\hat{\pi}_{1}$(**in bold**) $\hat{\pi}_{2}$ with respect to $\hat{\pi}_{SG}$ $n_{1}=r_{1}=1$,$n_{2}=r_{2}=6$, $c_{1}=c_{2}=1$, $a_{1}=9$, $b_{1}=3$,$a_{2}=10$, $b_{2}=4$, $\theta_{1}^{*}=0.7$.

| $\pi$ | | | | | | | | |
| --- | --- | --- | --- | --- | --- | --- | --- | --- |
| 0.1 | 0.2 | 0.3 | 0.4 | 0.5 | 0.6 | 0.7 | 0.8 | 0.9 |
| $\theta_{2}^{*}=0.2$ | | | | | | | | |
| **4.632** | **3.675** | **3.113** | **2.742** | **2.478** | **2.277** | **2.114** | **1.970** | **1.804** |
| 5.589 | 4.210 | 3.465 | 2.997 | 2.674 | 2.435 | 2.248 | 2.088 | 1.916 |
| $\theta_{2}^{*}=0.4$ | | | | | | | | |
| **7.904** | **6.144** | **5.124** | **4.467** | **4.020** | **3.713** | **3.521** | **3.464** | **3.741** |
| 9.537 | 7.039 | 5.703 | 4.881 | 4.338 | 3.972 | 3.743 | 3.671 | 3.974 |
| $\theta_{2}^{*}=0.6$ | | | | | | | | |
| **73.010** | **57.938** | **49.954** | **45.745** | **44.168** | **45.100** | **49.478** | **60.885** | **96.508** |
| 88.103 | 66.381 | 55.598 | 49.986 | 47.663 | 48.241 | 52.605 | 64.522 | 102.521 |
| $\theta_{2}^{*}=0.8$ | | | | | | | | |
| **42.036** | **38.110** | **37.327** | **38.682** | **42.167** | **48.569** | **60.135** | **83.669** | **150.461** |
| 50.726 | 43.664 | 41.544 | 42.268 | 45.503 | 51.952 | 63.936 | 88.667 | 159.835 |
