# Supplementary material for: General Randomized Response Techniques Using Polya's Urn Process as a Randomization Device
Source: PLoS One. 2014 Dec 26;9(12):e115612. doi: 10.1371/journal.pone.0115612 (PMC4277314; doi:10.1371/journal.pone.0115612)
Supplement: S9 Table — Relative efficiency of (in bold) with respect to for , , , , , , , . (DOCX) [file pone.0115612.s009.docx]

**Table S9:** Relative efficiency of $\hat{\pi}_{1}$(**in bold**) $\hat{\pi}_{2}$ with respect to $\hat{\pi}_{SG}$ $n_{1}=r_{1}=1$,$n_{2}=r_{2}=6$, $c_{1}=c_{2}=1$, $a_{1}=9$, $b_{1}=3$,$a_{2}=10$, $b_{2}=4$, $\theta_{1}^{*}=0.9$.

| $\pi$ | | | | | | | | |
| --- | --- | --- | --- | --- | --- | --- | --- | --- |
| 0.1 | 0.2 | 0.3 | 0.4 | 0.5 | 0.6 | 0.7 | 0.8 | 0.9 |
| $\theta_{2}^{*}=0.2$ | | | | | | | | |
| **4.972** | **3.926** | **3.311** | **2.906** | **2.617** | **2.397** | **2.220** | **2.063** | **1.883** |
| 6.000 | 4.498 | 3.685 | 3.175 | 2.824 | 2.564 | 2.360 | 2.186 | 2.000 |
| $\theta_{2}^{*}=0.4$ | | | | | | | | |
| **7.165** | **5.562** | **4.626** | **4.014** | **3.585** | **3.271** | **3.038** | **2.872** | **2.804** |
| 8.646 | 6.373 | 5.149 | 4.386 | 3.869 | 3.499 | 3.230 | 3.043 | 2.979 |
| $\theta_{2}^{*}=0.6$ | | | | | | | | |
| **13.080** | **10.043** | **8.292** | **7.176** | **6.432** | **5.947** | **5.691** | **5.744** | **6.642** |
| 15.785 | 11.506 | 9.228 | 7.841 | 6.941 | 6.361 | 6.051 | 6.087 | 7.055 |
| $\theta_{2}^{*}=0.8$ | | | | | | | | |
| **59.425** | **46.350** | **39.189** | **35.094** | **33.019** | **32.710** | **34.617** | **40.782** | **61.250** |
| 71.710 | 53.105 | 43.617 | 38.347 | 35.631 | 34.988 | 36.804 | 43.217 | 65.067 |
