# Supplementary material for: General Randomized Response Techniques Using Polya's Urn Process as a Randomization Device
Source: PLoS One. 2014 Dec 26;9(12):e115612. doi: 10.1371/journal.pone.0115612 (PMC4277314; doi:10.1371/journal.pone.0115612)
Supplement: S10 Table — Relative efficiency of (in bold) with respect to for , , , , , , , . (DOCX) [file pone.0115612.s010.docx]

**Table S10:** Relative efficiency of $\hat{\pi}_{1}$(**in bold**) $\hat{\pi}_{2}$ with respect to $\hat{\pi}_{SG}$ $n_{1}=r_{1}=1$,$n_{2}=r_{2}=6$, $c_{1}=c_{2}=-1$, $a_{1}=9$, $b_{1}=3$,$a_{2}=10$, $b_{2}=4$, $\theta_{1}^{*}=0.1$.

| $\theta_{2}^{*}=0.2$ | | | | | | | | |
| --- | --- | --- | --- | --- | --- | --- | --- | --- |
| **7.455** | **6.872** | **6.971** | **7.458** | **8.337** | **9.781** | **12.257** | **17.154** | **30.784** |
| 5.480 | 5.605 | 5.990 | 6.616 | 7.558 | 9.012 | 11.446 | 16.232 | 29.702 |
| $\theta_{2}^{*}=0.4$ | | | | | | | | |
| **1.975** | **2.411** | **2.776** | **3.176** | **3.687** | **4.411** | **5.571** | **7.791** | **13.873** |
| 1.452 | 1.967 | 2.385 | 2.818 | 3.342 | 4.064 | 5.203 | 7.372 | 13.385 |
| $\theta_{2}^{*}=0.6$ | | | | | | | | |
| **1.491** | **1.953** | **2.298** | **2.652** | **3.083** | **3.682** | **4.630** | **6.433** | **11.357** |
| 1.096 | 1.593 | 1.975 | 2.352 | 2.795 | 3.393 | 4.324 | 6.087 | 10.958 |
| $\theta_{2}^{*}=0.8$ | | | | | | | | |
| **1.346** | **1.801** | **2.131** | **2.461** | **2.858** | **3.405** | **4.268** | **5.903** | **10.367** |
| 0.989 | 1.469 | 1.831 | 2.183 | 2.591 | 3.137 | 3.985 | 5.586 | 10.003 |
