# Supplementary material for: General Randomized Response Techniques Using Polya's Urn Process as a Randomization Device
Source: PLoS One. 2014 Dec 26;9(12):e115612. doi: 10.1371/journal.pone.0115612 (PMC4277314; doi:10.1371/journal.pone.0115612)
Supplement: S11 Table — Data obtained through proposed RRT 1 using , , , , , . (DOC) [file pone.0115612.s011.doc]

**Table: S11:** Data obtained through proposed RRT 1 using $n_{2}=n_{2}=3$, $c_{1}=c_{2}=1$, $a_{1}=1000$, $b_{1}=30$,$a_{2}=100$, $b_{2}=40$.

| 2 | 3 | 2 | 2 | 3 | 3 | 3 | 3 | 2 | 3 | 1 | 0 | 2 | 3 | 3 | 3 | 3 | 3 | 3 | 2 | 1 | 2 | 3 | 2 | 2 |
| --- | --- | --- | --- | --- | --- | --- | --- | --- | --- | --- | --- | --- | --- | --- | --- | --- | --- | --- | --- | --- | --- | --- | --- | --- |
| 3 | 0 | 3 | 2 | 2 | 2 | 3 | 3 | 2 | 3 | 3 | 2 | 3 | 3 | 2 | 2 | 2 | 2 | 3 | 2 | 3 | 2 | 2 | 2 | 2 |
| 3 | 2 | 3 | 2 | 2 | 2 | 3 | 2 | 3 | 3 | 2 | 3 | 1 | 2 | 3 | 3 | 3 | 2 | 2 | 3 | 1 | 1 | 2 | 1 | 2 |
| 1 | 3 | 3 | 2 | 2 | 3 | 2 | 1 | 2 | 2 | 2 | 2 | 3 | 2 | 3 | 3 | 3 | 3 | 3 | 3 | 3 | 1 | 1 | 1 | 3 |
