# Supplementary material for: General Randomized Response Techniques Using Polya's Urn Process as a Randomization Device
Source: PLoS One. 2014 Dec 26;9(12):e115612. doi: 10.1371/journal.pone.0115612 (PMC4277314; doi:10.1371/journal.pone.0115612)
Supplement: S13 Table — Data obtained through [2] RRT using , . (DOCX) [file pone.0115612.s013.docx]

**Table: S13:** Data obtained through [2] RRT using $\theta_{1}^{*}=0.3$, $\theta_{2}^{*}=0.7$

| 0 | 0 | 0 | 4 | 1 | 2 | 3 | 0 | 2 | 6 | 0 | 2 | 7 | 0 | 1 | 4 | 0 | 1 | 3 | 1 | 0 | 7 | 1 | 1 | 1 |
| --- | --- | --- | --- | --- | --- | --- | --- | --- | --- | --- | --- | --- | --- | --- | --- | --- | --- | --- | --- | --- | --- | --- | --- | --- |
| 0 | 1 | 2 | 8 | 0 | 1 | 1 | 4 | 0 | 1 | 2 | 0 | 9 | 0 | 0 | 0 | 1 | 0 | 2 | 1 | 3 | 0 | 2 | 6 | 4 |
| 0 | 2 | 1 | 0 | 7 | 8 | 1 | 0 | 0 | 0 | 1 | 1 | 2 | 0 | 1 | 0 | 1 | 0 | 1 | 0 | 4 | 1 | 1 | 1 | 0 |
| 4 | 1 | 0 | 12 | 2 | 1 | 1 | 1 | 0 | 0 | 1 | 1 | 2 | 9 | 3 | 1 | 1 | 9 | 1 | 3 | 0 | 2 | 3 | 4 | 0 |
